# Editorial: Shiga Toxin-Converting Bacteriophages

**DOI:** 10.3389/fmicb.2021.680816

**Published:** 2021-05-04

**Authors:** Grzegorz Węgrzyn, Maite Muniesa

**Affiliations:** ^1^Department of Molecular Biology, Faculty of Biology, University of Gdansk, Gdansk, Poland; ^2^Department of Genetics, Microbiology and Statistics, University of Barcelona, Barcelona, Spain

**Keywords:** Shiga toxin, bacteriophages, enterohemorrhagic *Escherichia coli*, phage development, phage evolution

Shiga toxin-producing *Escherichia coli* (STEC) strains, and particularly a subset of this group of bacteria called enterohemorrhagic *E. coli* (EHEC), are dangerous human pathogens (Newell and La Ragione, 2018). They cause bloody diarrhea and some other symptoms, but the most severe complication, causing the death of a relatively large fraction of patients, is hemolytic uremic syndrome (Karmali, 2018; Joseph et al., 2020). The major virulence factors of STEC are Shiga toxins that are complex proteins (composed of one A and five B subunits; AB_5_) which enter an eukaryotic cell mainly through the interaction of the toxin B subunit with the Gb3 and/or Gb4 receptor. After endocytosis, followed by retro-translocation from the Golgi apparatus and endoplasmic reticulum, the toxin A subunit, which is an actual toxin, is released into the cytoplasm where it inactivates ribosomes by specific cleavage of one of the adenine residues in 28S rRNA (Menge, 2020).

The crucial point of virulence of STEC is the fact that Shiga toxins are not encoded in *E. coli* chromosomes but their genes, called *stx* genes, are located in the genomes of bacteriophages which occur in their hosts as prophages (Loś et al., 2011). These bacteriophages, called also Stx phages, belong to the group of lambdoid phages. After infection, Stx phages may lysogenize the host cell. Following integration of the phage DNA into the bacterial chromosome, it can be passively replicated together with the host genome. In lysogenic cells, the vast majority of phage genes are silent, including *stx* genes. Under conditions causing the appearance of single-stranded DNA fragments which induce the bacterial S.O.S. response, the phage-encoded major repressor (the cI protein) is cleaved. This leads to induction of the prophage, excision of phage DNA, and subsequent lytic development which consists of effective viral DNA replication, production of progeny virions, and their release after host cell lysis. This is accompanied with massive production and liberation of Shiga toxins. Although under laboratory conditions, UV irradiation or DNA-binding antibiotics, like mitomycin C, are routinely used to activate the S.O.S. response, it appears that oxidative stress may be one of major causes of Stx prophage induction in STEC-infected humans (Licznerska et al., 2016). Importantly, contrary to early predictions of uniformity in Stx phages, this group of viruses has been recognized as highly variable (Krüger and Lucchesi, 2015). An *E. coli* host can be lysogenized not only by one, but also by two or more Stx phages which may result in the production of different types of Stx toxins in one bacterial cell. In fact, it was demonstrated that understanding the biodiversity and evolution of Stx phages may have a great impact on both the accurate diagnostics of STEC-mediated infections and development of novel therapeutic approaches (Martínez-Castillo and Muniesa, 2014).

The treatment of STEC-infected patients is particularly difficult as it was found that many antibiotics are potent inducers of prophages. Therefore, even if antibiotic treatment can kill bacterial cells or inhibit their growth, prophage induction would cause effective production of Shiga toxin in the meantime which could be more dangerous for patients than the presence of STEC cells. Although some antibiotics can kill bacteria without stimulating the S.O.S. response, the use of antibiotics in the treatment of STEC infections remains controversial (Bielaszewska et al., 2012). Such a medical procedure is formally forbidden in some countries if this type of disease is confirmed or even suspected (Kakoullis et al., 2019). In this light, development of alternative therapies appears crucial (Mühlen and Dersch, 2020). On the other hand, effective management of diseases caused by STEC requires an understanding of the details of pathogenicity mechanisms and epidemiology, and development of rapid and accurate diagnostic methods (Paletta et al., 2020). Cattle are considered to be the main reservoir of STEC (Kim et al., 2020). This is because bovine vascular cells do not contain the Gb3 receptor [though it may be present in some other tissues, as reported by Pruimboom-Brees et al. (2000)], therefore, Shiga toxins cannot enter them efficiently (Sapountzis et al., 2020). Nevertheless, despite enormous progress in studies on various aspects of STEC and Stx phages, it appears that we are still far from understanding the details of their diversity, evolution, distribution, and molecular mechanisms of functioning.

In the light of the problems and open questions summarized above, a special issue of this journal has been devoted to present recent advances in studies on Stx phages. According to recent trends, articles published in this issue are focused on biodiversity and evolution of these phages, as well as on molecular mechanisms of their development, including genetic recombination, which seems to be of particular importance in increasing variability of viruses (general recombination) and virulence of *E. coli* strains (site-specific recombination).

A novel view on evolution of Stx phages has been presented by Zuppi et al. They have isolated newly discovered Stx phages from STEC strains, analyzed phages' whole-genome sequences and compared them to previously sequenced genomes of other Stx phages. Considerable variability of these phages was noted, and multiple transduction events have been predicted. The crucial conclusion from this study was that “colonization of a specific reservoir by STEC strains could be influenced by the Stx phage that they carry.” This is an important contribution to our understanding of the distribution of STEC in animal carriers, and further attempts to recognize reservoirs of these bacteria more accurately. Genomic diversity of Stx phages has been indicated also in another article, by Zhang et al. Newly identified bacteriophages have been characterized after prophage induction from environmental STEC strains. This study provided further important evidence for Shiga toxin conversion among *E. coli* strains through Stx phage lysogenic infection. In this light, it appears crucial to estimate the efficiency of survival and persistence of Stx phages in food products, since they can spread virulence factors under favorable conditions, as stated by Spilsberg et al. These authors described an improved procedure for the detection of Stx phages in minced meat, employing real-time PCR and digital droplet PCR. Using this method, they demonstrated that these phages could survive and remain infective for at least 20 days during meat storage.

Recombination events may significantly contribute to the variability of Stx phages, and the article by Greig et al. provides examples of the occurrence of such processes. Definitely, evolutionary analyses of Stx phages and STEC strains should take into consideration genetic recombination. The authors concluded that when studying the persistence and survival of STEC in the environment, microevolutionary processes and larger changes in genomes should be analyzed by using both short and long read technologies, in order to understand their mechanisms in more detail. The importance of such a research strategy has been underlined in another study by Greig et al. in which they analyzed biological material derived from a STEC-caused outbreak of foodborne disease. Epidemiological data suggested that the outbreak strains originated from one country while phylogenetic studies indicated that they were most related to isolates from geographically very distinct regions. As proposed by the authors, early warning of emerging threats to public health can be facilitated by monitoring transmission of STEC and Stx phages.

As indicated above, Stx phages belong to lambdoid bacteriophages, as classified on the basis of the organization of their genomes. However, two articles published in this special issue indicated that the molecular mechanisms of development of various Stx phages might be very different from those found in bacteriophage λ. Mohaisen et al. studied the site-specific recombination system of the Stx bacteriophage ϕ24_B_ and found that *att* regions in genomes of this phage and its host differ significantly from those occurring in λ and between *gal* and *bio* loci in *E. coli* chromosomes. Moreover, the integration of the ϕ24_B_ genome appeared independent on the integration host factor (the IHF protein), contrary to the strict dependence on IHF in the case of analogous reaction in λ. Similarly, a DNA replication system of Eru bacteriophages (a newly identified group of Stx phages), which is completely unrelated to that of λ, has been described by Llarena et al. The authors proposed a novel classification of Stx phages, based on their replication regions, in addition to the type of *stx* genes they carry.

In summary, recent studies have brough unexpected and fascinating results which are important to understand the biology of Stx phages in more detail. Therefore, we encourage researchers to read the articles included in this special issue, and to contribute to our collection of knowledge on these important viruses.

## Author Contributions

GW and MM contributed to the analysis of the reviewed articles and to preparing the manuscript.

## Conflict of Interest

The authors declare that the research was conducted in the absence of any commercial or financial relationships that could be construed as a potential conflict of interest.

## References

[B1] Bielaszewska M. Idelevich E. A. Zhang W. Bauwens A. Schaumburg F. Mellmann A. Peters G. Karch H. (2012). Effects of antibiotics on Shiga toxin 2 production and bacteriophage induction by epidemic *Escherichia coli* O104:H4 strain. Antimicrob. Agents Chemother. 56, 3277–3282. 10.1128/AAC.06315-11 22391549 PMC3370775

[B2] Joseph A. Cointe A. Mariani Kurkdjian P. Rafat C. Hertig A. (2020). Shiga toxin-associated hemolytic uremic syndrome: a narrative review. Toxins 12:67. 10.3390/toxins12020067 31973203 PMC7076748

[B3] Kakoullis L. Papachristodoulou E. Chra P. Panos G. (2019). Shiga toxin-induced haemolytic uraemic syndrome and the role of antibiotics: a global overview. J. Infect. 79, 75–94. 10.1016/j.jinf.2019.05.018 31150744

[B4] Karmali M. A. (2018). Factors in the emergence of serious human infections associated with highly pathogenic strains of Shiga toxin-producing *Escherichia coli*. Int. J. Med. Microbiol. 308, 1067–1072. 10.1016/j.ijmm.2018.08.005 30146439

[B5] Kim J. S. Lee M. S. Kim J. H. (2020). Recent updates on outbreaks of Shiga toxin-producing *Escherichia coli* and its potential reservoirs. Front. Cell Infect. Microbiol. 10:273. 10.3389/fcimb.2020.00273 32582571 PMC7287036

[B6] Krüger A. Lucchesi P. M. (2015). Shiga toxins and stx phages: highly diverse entities. Microbiology 161, 451–462. 10.1099/mic.0.000003 25479836

[B7] Licznerska K. Nejman-Faleńczyk B. Bloch S. Dydecka A. Topka G. Gasior T.. (2016). Oxidative stress in Shiga toxin production by enterohemorrhagic *Escherichia coli*. Oxid. Med. Cell. Longev. 2016:3578368. 10.1155/2016/3578368 26798420 PMC4699097

[B8] Loś J. M. Loś M. Wegrzyn G. (2011). Bacteriophages carrying shiga toxin genes: genomic variations, detection and potential treatment of pathogenic bacteria. Future Microbiol. 6, 909–924. 10.2217/fmb.11.70 21861621

[B9] Martínez-Castillo A. Muniesa M. (2014). Implications of free Shiga toxin-converting bacteriophages occurring outside bacteria for the evolution and the detection of Shiga toxin-producing *Escherichia coli*. Front. Cell. Infect. Microbiol. 4:46. 10.3389/fcimb.2014.00046 24795866 PMC3997033

[B10] Menge C. (2020). Molecular biology of *Escherichia coli* Shiga toxins' effects on mammalian cells. Toxins 12:345. 10.3390/toxins12050345 32456125 PMC7290813

[B11] Mühlen S. Dersch P. (2020). Treatment strategies for infections with Shiga toxin-producing *Escherichia coli*. Front. Cell. Infect. Microbiol. 10:169. 10.3389/fcimb.2020.00169 32435624 PMC7218068

[B12] Newell D. G. La Ragione R. M. (2018). Enterohaemorrhagic and other Shiga toxin-producing *Escherichia coli* (STEC): where are we now regarding diagnostics and control strategies? Transbound. Emerg. Dis. 65, 49–71. 10.1111/tbed.12789 29369531

[B13] Paletta A. C. C. Castro V. S. Conte-Junior C. A. (2020). Shiga toxin-producing and enteroaggregative *Escherichia coli* in animal, foods, and humans: pathogenicity mechanisms, detection methods, and epidemiology. Curr. Microbiol. 77, 612–620. 10.1007/s00284-019-01842-1 31834432

[B14] Pruimboom-Brees I. M. Morgan T. W. Ackermann M. R. Nystrom E. D. Samuel J. E. Cornick N. A.. (2000). Cattle lack vascular receptors for *Escherichia coli* O157:H7 shiga toxins. Proc. Natl. Acad. Sci. U.S.A. 97, 10325–10329. 10.1073/pnas.190329997 10973498 PMC27023

[B15] Sapountzis P. Segura A. Desvaux M. Forano E. (2020). An overview of the elusive passenger in the gastrointestinal tract of cattle: the shiga toxin producing *Escherichia coli*. Microorganisms 8:877. 10.3390/microorganisms8060877 32531983 PMC7355788

